# Uterine Myxoid Mesenchymal Tumor With a Novel *SS18::VEZF1* Gene Fusion, Lacking Worrisome Histological Features

**DOI:** 10.1002/gcc.70079

**Published:** 2025-08-28

**Authors:** Isidro Machado, Reyes Claramunt, Susana López, Jessica Aliaga, Enrique Garrigós, Isabel Martín, Ignacio Romero, Antonio Llombart‐Bosch, José Antonio López‐Guerrero

**Affiliations:** ^1^ Pathology Department Instituto Valenciano de Oncología Valencia Spain; ^2^ Patologika Laboratory Hospital QuironSalud Valencia Spain; ^3^ Pathology Department University of Valencia Valencia Spain; ^4^ CIBERONC Madrid Spain; ^5^ Molecular Biology Laboratory Instituto Valenciano de Oncología Valencia Spain; ^6^ Gynecology Department Instituto Valenciano de Oncología Valencia Spain; ^7^ Radiology Department Instituto Valenciano de Oncología Valencia Spain; ^8^ Oncology Department Instituto Valenciano de Oncología Valencia Spain; ^9^ Pathology Department Universidad Católica Valencia Spain; ^10^ Joint Cancer Research Unit IVO‐CIPF Valencia Spain

**Keywords:** *SS18* rearrangement, *SS18::VEZF1* gene fusion, uterine myxoid mesenchymal tumor

## Abstract

We report a uterine myxoid mesenchymal tumor with a novel *SS18::VEZF1* gene fusion. The current lesion was identified in a 53‐year‐old woman who presented with symptomatic “fibroids” showing accelerated growth and heterogeneous morphology on radiologic assessment. Microscopic examination revealed a well‐demarcated neoplasm, and the tumor exhibited alternating hypocellular/hyalinized and hypercellular areas, composed of a monomorphic proliferation of spindle, ovoid, and epithelioid cells arranged in sheets. These cells were embedded within either a hyalinized collagenous stroma or abundant myxoid stroma. Tumor cells were frequently located around blood vessels and exhibited amphophilic or eosinophilic cytoplasm and elongated or ovoid‐shaped nuclei with coarsely clumped chromatin. No mitoses, pleomorphism, or necrosis was identified. Immunohistochemically, the tumor was positive for CD10, CD34, TLE1, estrogen, and progesterone receptors. It was negative for h‐caldesmon, desmin, smooth muscle actin, smoothelin, myosin, cyclin D1, S100, ALK, EMA, panTRK, and SS18‐SSX. Targeted RNA sequencing revealed an *SS18::VEZF1* gene fusion (breakpoint: *exon 9*–*exon 2*), which was confirmed by FISH (*SS18*). In conclusion, RNA sequencing was useful in identifying the fusion event, thereby excluding potential mimics with uncommon morphology or ambiguous immunophenotype.

## 1 Introduction

The category of uterine myxoid neoplasms remains diagnostically challenging and is still poorly understood from both clinical and biological perspectives [1, 2, 3, 4, 5]. These tumors frequently harbor gene fusions, making molecular testing a valuable tool in their diagnostic evaluation, especially given the significant morphologic and immunohistochemical overlap among various entities [1, 2, 3, 4, 5].

We report a case of uterine myxoid mesenchymal neoplasm characterized by bland histologic features and an *SS18::VEZF1* fusion. Although this is a recent case and long‐term follow‐up data are not yet available, we consider it noteworthy that the *SS18::VEZF1* fusion may occur in tumors with apparently benign morphology and a predominant stromal endometrial phenotype, rather than a smooth muscle phenotype.

## 2 Materials and Methods

In the clinical work‐up of a uterine tumor with predominant myxoid/hyalinized stromal tissue, targeted RNA sequencing (RNA‐seq) was performed for a more definitive subclassification due to unusual and conflicting histologic findings and immunoprofile. Informed consent was obtained from the patient reported in this study.

### 2.1 Immunohistochemistry

Immunohistochemical stains, including their sources, clones, and dilutions, are described in Table S1. All positive and negative controls showed appropriate staining.

### 2.2 RNA‐seq

RNA was extracted from formalin‐fixed, paraffin‐embedded (FFPE) tissue using the RecoverAll Total Nucleic Acid Isolation Kit for FFPE (Invitrogen, Thermo Fisher Scientific Inc., Waltham, Massachusetts, USA), following the manufacturer's instructions. The FusionPlex Sarcoma v2 panel (ArcherDX, Boulder, CO, USA) was used to perform a targeted RNA panel, also according to the manufacturer's protocol. The FusionPlex Sarcoma v2 panel is an optimized, balanced pool of gene‐specific primer (GSP) oligonucleotides that is used in conjunction with FusionPlex Reagents and Molecular Barcode (MBC) Adapters to produce targeted NGS libraries. FusionPlex Sarcoma v2 contains 659 GSPs targeting 63 genes commonly altered in sarcomas.

Sequencing was performed on the Illumina NextSeq 550 System (Illumina, San Diego, CA), and data analysis was conducted using Archer Analysis software (Archer Analysis 7.3). The Archer Analysis software utilizes these MBCs for error correction and reads deduplication to support quantitative multiplex data analysis and confident variant detection. Archer Analysis reports both sequencing metrics and the number of unique observations supporting called variants.

### 2.3 Fluorescence In Situ Hybridization (FISH)

FFPE section tissue was used to perform. We used the SPEC SS18 Dual Color Break‐Apart Probe (ZytoVision GmbH, Germany). At least a total of 100 nuclei were counted with the assistance of two independent observers. *SS18* rearrangement was interpreted based on the presence of a predominant atypical signal pattern with extra‐signal *SS18* 5′ in more than10%–15% of tumor cell nuclei and an isolated break‐apart pattern.

## 3 Results

### 3.1 Case Report

**FIGURE 1:**
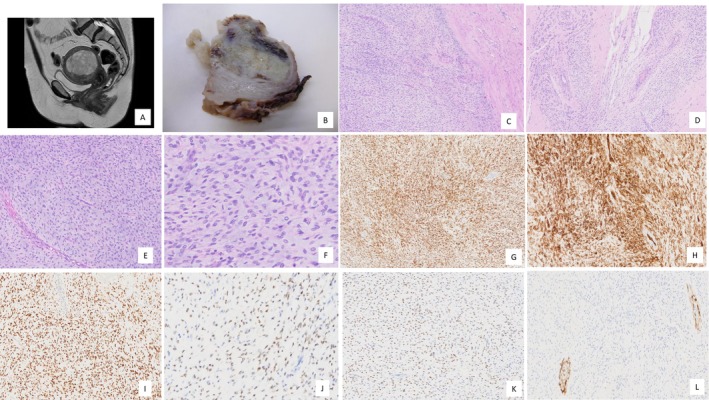
(A) Magnetic resonance imaging shows a well‐defined, intramural, multilobulated, and heterogeneous uterine lesion. (B) The fragmented surgical specimen contains a well‐demarcated, nodular myxoid lesion. (C) Histopathological examination with hematoxylin and eosin (H&E) reveals a well‐demarcated myxoid mesenchymal neoplasm without necrosis (original magnification ×10). (D) The tumor exhibits alternating hypocellular/hyalinized and hypercellular areas. Neoplastic cells are frequently located around blood vessels, occasionally showing a concentric perivascular growth pattern (H&E, ×10). (E) The tumor demonstrates hypercellular areas composed of a monomorphic proliferation of spindle and ovoid cells arranged in sheets, with blood vessels in the background featuring variable wall thickness (H&E, ×20). (F) The neoplastic cells display amphophilic to eosinophilic cytoplasm and spindle to ovoid nuclei with coarsely clumped chromatin in a predominantly myxoid background. No mitoses or necrosis are observed (H&E, ×40). (G, H) Strong and diffuse CD10 and CD34 immunoreactivity (×40). (I) Strong and diffuse nuclear expression of progesterone receptor (×10). (J) Patchy estrogen receptor expression in tumor cells (×20). (K) Moderate and patchy TLE1 immunoexpression in tumor cells (×20). (L) H‐Caldesmon negative in tumor cells, with positive internal control (×10).

The patient, a 53‐year‐old woman with no significant prior medical history, presented to our institution with symptomatic uterine “fibroids,” one of which had demonstrated rapid growth. Diagnostic MRI revealed a well‐defined, intramural, multilobulated, and heterogeneous uterine lesion measuring 69 mm in its largest dimension (Figure 1A). The radiologist noted that the lesion did not display typical features of a myoma and recommended either close follow‐up or surgical removal.

A vaginal hysterectomy with morcellation was subsequently performed. The surgical specimen was received in a fragmented state and contained a nodular, myxoid lesion measuring 85 mm in its largest dimension (Figure 1B). No areas of necrosis were identified. Histopathological examination revealed a well‐demarcated neoplasm distinct from the normal uterine smooth muscle (Figure 1C). The tumor exhibited alternating hypocellular/hyalinized and hypercellular areas (Figure 1D), composed of a monomorphic proliferation of spindle, ovoid, and epithelioid cells arranged in sheets, with blood vessels in the background featuring variable wall thickness (Figure 1E,F). These cells were embedded within a hyalinized collagenous stroma, with abundant myxoid stromal tissue present in most areas (Figure 1E,F). No infiltrative growth pattern was observed. Tumor cells were frequently located around blood vessels, occasionally displaying a whirling or concentric perivascular growth pattern (Figure 1D). Additionally, the neoplastic cells exhibited amphophilic or eosinophilic cytoplasm and elongated to ovoid‐shaped nuclei with coarsely clumped chromatin and occasional conspicuous nucleoli (Figure 1F). The mitotic rate was low, with fewer than one mitosis per 10 high‐power fields. No nuclear hyperchromasia, pleomorphism, significant atypia, or necrosis was identified. Immunohistochemically, the neoplastic cells were positive for CD10, CD34 (Figure 1G,H), progesterone receptor (Figure 1I), estrogen receptor (Figure 1J), and TLE1 (Figure 1K). They were negative for h‐caldesmon (Figure 1L), desmin, smooth muscle actin, smoothelin, myosin, S100, PanTRK, ALK, EMA, SS18‐SSX, and cyclin D1.

**FIGURE 2:**
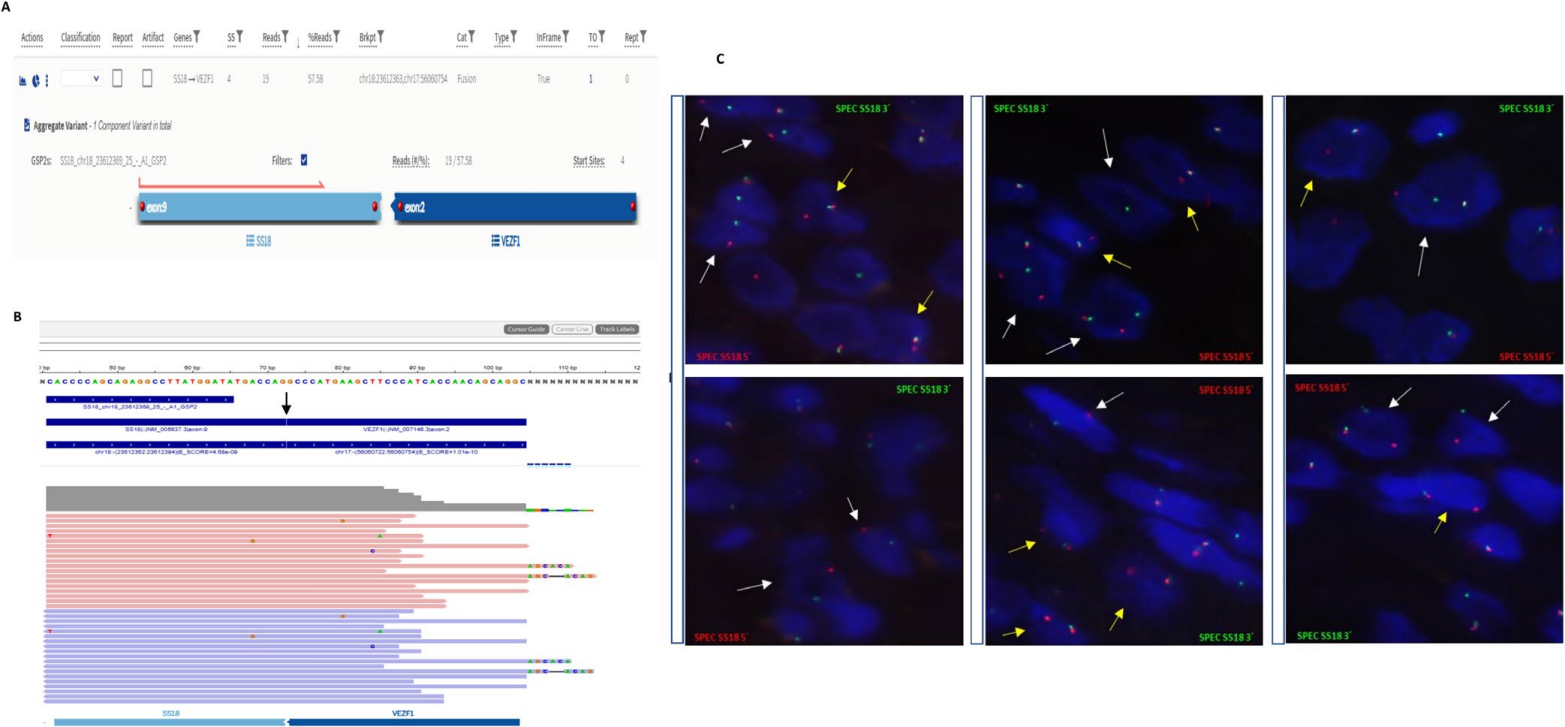
(A) Screenshot from Archer Analysis software version 7.2.0‐1 showing the *SS18::VEZF1* gene fusion identified by next‐generation sequencing (NGS). (B) Integrative Genomics Viewer (IGV) displaying the *SS18::VEZF1* gene fusion. Breakpoints are indicated by black arrows. (C) FISH analysis of the SS18 (subunit of BAF chromatin remodeling complex) gene using a break‐apart probe. The images display various patterns of positive (rearranged) signals for the *SS18* gene. One positive signal pattern corresponds to an atypical pattern characterized by a single red signal (SS18 5′) and a normal nucleus with overlapping red (SS18 5′) and green (SS18 3′) signals, indicated by yellow arrows. The second positive signal pattern corresponds to a typical rearranged pattern, with separated green (SS18 3′) and red (SS18 5′) signals, indicated by white arrows.

A provisional diagnosis of a myxoid mesenchymal uterine tumor was made. RNA‐seq using the Archer FusionPlex Sarcoma v2 panel revealed an *SS18 (exon9)::VEZF1(exon2)* gene fusion in 19 reads (number of unique reads supporting the event) (Figure 2A,B), which was further confirmed by FISH. A rearrangement of *SS18* was observed in 77% of nuclei analyzed (Figure 2C). These findings supported the final classification of the tumor as a uterine myxoid mesenchymal neoplasm harboring an *SS18::VEZF1* fusion.

## 4 Discussion

The *SS18* gene, located at chromosome 18q11.2, plays essential roles in both normal development and oncogenesis, especially in synovial sarcoma [6]. The canonical fusions involving this gene are *SS18::SSX1, SS18::SSX2*, or *SS18::SSX4* found in the vast majority of synovial sarcomas [6]. Beyond these, several *non‐SSX* fusion partners have also been reported (*CRTC1*; *POU5F1*; *NEDD4*; *MEF2D*; *MEF2C*; *ZBTB7A* and *GREB1*) [7, 8, 9, 10] and these variant fusions span a diverse range of tumor types including extra‐uterine and uterine sarcomas, adenocarcinomas, and hematologic malignancies, highlighting the broad oncogenic potential of *SS18* rearrangements [7, 8, 9, 10].

Vascular endothelial zinc finger 1 (*VEZF1*) is a gene located on chromosome 17 at position q22. It encodes a transcription factor that is primarily expressed in endothelial cells and plays a crucial role in blood vessel development and function [11]. Although it contributes to angiogenesis in healthy tissue, VEZF1 can also function as an oncoprotein in certain cancers, promoting tumor growth and metastasis, as observed in hepatocellular carcinoma [11].

To date, the *EWSR1::VEZF1* fusion has been reported in a malignant peripheral nerve sheath tumor (MPNST) with heterologous differentiation [12] and in an unclassified round cell sarcoma [13]. Additionally, the *SS18::VEZF1* fusion has been identified in one unclassified uterine tumor within a large series of 609 human tumors tested using an SS18‐SSX antibody; however, the authors did not report the clinicopathological features of this case [14].

In 2021, Hodgson et al. presented two cases of uterine myxoid sarcomas harboring the *SS18::VEZF1* fusion (breakpoint exon10–exon2) at the USCAP meeting (the abstract form is available only) [15]. Both cases exhibited concerning clinical or histologic features—specifically, lymphovascular invasion in case 1 and recurrence in case 2 [15]. Although both reported cases in USCAP displayed myxoid stroma, they also showed hybrid features of smooth muscle and endometrial stromal neoplasia [15], which contrasts with our present case. Our case lacks smooth muscle differentiation, as confirmed by multiple immunohistochemical stains and demonstrates an immunophenotype more consistent with low‐grade endometrial stromal neoplasia.

Tumors with a predominantly myxoid stroma are uncommon in the uterus; when present, however, they pose a significant diagnostic challenge [1, 2, 3, 4, 5, 16, 17, 18, 19, 20, 21, 22, 23, 24, 25]. Several uterine neoplasms may display a myxoid pattern, including myxoid leiomyoma/leiomyosarcoma [1, 2, 3, 4, 5], inflammatory myofibroblastic tumor (IMT) [1, 2, 3, 4, 5, 17], low‐ and high‐grade endometrial stromal sarcoma (ESS) [2, 3, 4, 5, 18, 19], *NTRK*‐rearranged neoplasms [20, 21], *PLAG1*‐altered uterine tumors [22], uterine sarcoma harboring a *KAT6B::KANSL1* fusion [23], *NR4A3*‐rearranged gynecologic leiomyosarcomas [24], and *GLI1*‐altered tumors [25]. The detection of a single, specific genetic alteration can yield crucial diagnostic, predictive, prognostic, or therapeutic insights.

The diagnosis of myxoid leiomyoma or myxoid leiomyosarcoma relies on morphological assessment supported by immunohistochemistry [1, 2, 3, 4, 5]. These tumors characteristically express estrogen and progesterone receptors, and they demonstrate smooth muscle differentiation, with h‐caldesmon serving as the most specific marker [1, 2, 3, 4, 5]. Key worrisome features favoring a diagnosis of myxoid leiomyosarcoma include marked cytologic atypia, tumor cell necrosis, or more than 1–2 mitoses per 10 HPF in the absence of atypia or necrosis, particularly when margins are infiltrative [1, 2, 3, 4, 5].

IMT commonly exhibit a dense lymphoplasmacytic inflammatory infiltrate, are often ALK positive, and may harbor *ALK* or *ROS1* gene rearrangements [3, 4, 5, 17]. Expression of smooth muscle markers and CD10 is variable [3, 4, 5, 17].

Low‐ or high‐grade EES with a myxoid component can be a challenging differential diagnosis [2, 3, 4, 5, 18, 19]. These tumors are typically CD10‐positive; in high‐grade ESS, BCOR and cyclin D1 are often positive [2, 3, 4, 5, 18, 19], while high‐grade ESS commonly lacks smooth muscle marker expression. Molecular profiling provides additional diagnostic clues in challenging cases [18, 19]. Low‐grade ESS is commonly associated with *JAZF1*, *YWHAE*, or *PHF1* gene alterations, while high‐grade ESS is often characterized by *BCOR* or *YWHAE* gene alterations [18, 19].

*NTRK* fusions define a uterine sarcoma subtype with fibrosarcoma‐like features, and reported cases are characterized by haphazard fascicles of spindle cells, occasionally accompanied by focal myxoid stromal tissue [20, 21]. Although our current case exhibited strong and diffuse CD34 expression, raising concern for an *NTRK*‐rearranged tumor, pan‐TRK staining was negative, making an *NTRK*‐rearranged tumor unlikely.

More recently, several novel molecular subsets of gynecologic sarcomas have been characterized, including *PLAG1*‐rearranged tumors [22], *KAT6B/A::KANSL1* fusion sarcomas [23], *NR4A3*‐rearranged leiomyosarcomas [24], and *GLI1*‐altered neoplasms in the gynecologic tract [25]. *PLAG1*‐rearranged sarcomas often exhibit prominently hyalinized stroma and may present areas mimicking myxoid liposarcoma. Although *PLAG1* gene fusions have been identified in a subset of uterine myxoid leiomyosarcomas, recent studies have documented new cases of *PLAG1*‐rearranged uterine sarcomas lacking myxoid morphology and/or expression of smooth muscle markers [22]. *KAT6B/A::KANSL1* fusion sarcomas typically display hybrid morphology combining features of endometrial stromal differentiation, often with fibroblastic or fibromyxoid areas and smooth muscle differentiation [23]. Some of these tumors may also reveal focal or extensive myxoid components [23]. *NR4A3*‐rearranged gynecologic leiomyosarcomas, frequently harboring *PGR::NR4A3* or related fusions, often contain abundant myxoid matrix and hemorrhage that create a pulmonary edema‐like pattern [24]. *GLI1*‐altered neoplasms of the gynecologic tract demonstrate nodular growth with solid architecture comprising bland epithelioid to ovoid–spindle cells in variably myxoid or hyalinized stroma [25]. Their immunoprofiles are inconsistent, and molecular testing is essential for accurate diagnosis [20, 21].

Undoubtedly, advances in molecular profiling have greatly improved our ability to identify these emerging or provisional gene‐fusion–driven sarcoma subtypes in gynecologic tumors, providing additional diagnostic, predictive, and therapeutic insights in otherwise challenging cases.

Herewith, we report for the first time the whole clinicopathologic features of one uterine myxoid mesenchymal neoplasm with *SS18::VEZF1* fusion. While the molecular profile and gene fusion are consistent with the two previously presented cases of uterine myxoid sarcoma at the USCAP meeting [15], our case lacked histologic features suggestive of aggressive behavior.

In conclusion, RNA‐seq in this case was instrumental in identifying the fusion event, thereby excluding potential mimics. Additionally, it broadened the spectrum of tumors harboring this fusion, particularly those with ambiguous immunophenotypes. However, given that the biological spectrum of uterine tumors harboring this fusion remains poorly understood, the diagnosis, descriptive as it may be, continues to rely primarily on morphological assessment. Larger case series are needed to better define the incidence of the *SS18::VEZF1* fusion in both uterine and extrauterine tumors, to determine whether it is restricted to myxoid neoplasms, and to clarify its potential prognostic significance.

## Author Contributions

Isidro Machado, Reyes Claramunt, Susana López, José Antonio López‐Guerrero and Antonio Llombart‐Bosch contributed to the conception and design of the work, acquisition, and interpretation of data, drafting the MS, and revising it. All the authors have read and approved the final manuscript.

## Ethics Statement

This study was performed following IRB approval.

## Consent

Informed consent was obtained from the patient reported in this study.

## Conflicts of Interest

The authors declare no conflicts of interest.

## Supporting information


**Table S1:** Supporting Information.

## Data Availability

The data that support the findings of this study are available from the corresponding author upon reasonable request.

## References

[1] C. Parra‐Herran , J. K. Schoolmeester , L. Yuan , et al., “Myxoid Leiomyosarcoma of the Uterus: A Clinicopathologic Analysis of 30 Cases and Review of the Literature With Reappraisal of Its Distinction From Other Uterine Myxoid Mesenchymal Neoplasms,” American Journal of Surgical Pathology 40, no. 3 (2016): 285–301, 10.1097/PAS.0000000000000593. 26866354

[2] A. Busc and C. Parra‐Herran , “Myxoid Mesenchymal Tumors of the Uterus: An Update on Classification, Definitions, and Differential Diagnosis,” Advances in Anatomic Pathology 24, no. 6 (2017): 354–361, 10.1097/PAP.0000000000000164. 28787279

[3] S. Croce , M. Devouassoux‐Shisheboran , P. Pautier , et al., “Uterine Sarcomas and Rare Uterine Mesenchymal Tumors With Malignant Potential. Diagnostic Guidelines of the French Sarcoma Group and the Rare Gynecological Tumors Group,” Gynecologic Oncology 167, no. 2 (2022): 373–389, 10.1016/j.ygyno.2022.07.031. 36114030

[4] E. C. Kertowidjojo and J. A. Bennett , “Update on Uterine Mesenchymal Neoplasms,” Surgical Pathology Clinics 15, no. 2 (2022): 315–340, 10.1016/j.path.2022.02.008. 35715164

[5] J. Y. Yoon , A. Mariño‐Enriquez , N. Stickle , et al., “Myxoid Smooth Muscle Neoplasia of the Uterus: Comprehensive Analysis by Next‐Generation Sequencing and Nucleic Acid Hybridization,” Modern Pathology 32, no. 11 (2019): 1688–1697, 10.1038/s41379-019-0299-4. 31189997

[6] E. Baranov , M. J. McBride , A. M. Bellizzi , et al., “A Novel SS18‐SSX Fusion‐Specific Antibody for the Diagnosis of Synovial Sarcoma,” American Journal of Surgical Pathology 44, no. 7 (2020): 922–933, 10.1097/PAS.0000000000001447. 32141887 PMC7289668

[7] L. M. Warmke , S. A. Strike , L. M. Fayad , et al., “Undifferentiated Round Cell Sarcoma With CRTC1::SS18 Fusion: Expanding Clinicopathologic Features of a Rare Translocation Sarcoma With Prominent Desmoplastic Stroma,” Modern Pathology 37, no. 9 (2024): 100555, 10.1016/j.modpat.2024.100555. 38972355

[8] M. Zhang , D. Mao , and W. Zhang , “The Pathogenic Role of MEF2D‐SS18 Fusion Gene in B‐Cell Acute Lymphoblastic Leukemia,” Biochemical and Biophysical Research Communications 496, no. 4 (2018): 1331–1336, 10.1016/j.bbrc.2018.02.013. 29408457

[9] I. Weinreb , E. Hahn , B. C. Dickson , et al., “Microcribriform Adenocarcinoma of Salivary Glands: A Unique Tumor Entity Characterized by an SS18::ZBTB7A Fusion,” American Journal of Surgical Pathology 47, no. 2 (2023): 194–201, 10.1097/PAS.0000000000001980. 36221318

[10] C. H. Lee , Y. C. Kao , W. R. Lee , et al., “Clinicopathologic Characterization of GREB1‐Rearranged Uterine Sarcomas With Variable Sex‐Cord Differentiation,” American Journal of Surgical Pathology 43, no. 7 (2019): 928–942, 10.1097/PAS.0000000000001265. 31094921

[11] X. Shi , P. Zhao , and G. Zhao , “VEZF1, Destabilized by STUB1, Affects Cellular Growth and Metastasis of Hepatocellular Carcinoma by Transcriptionally Regulating PAQR4,” Cancer Gene Therapy 30, no. 2 (2023): 256–266, 10.1038/s41417-022-00540-8. 36241701

[12] S. Benini , G. Gamberi , S. Cocchi , et al., “Identification of a Novel Fusion Transcript EWSR1‐VEZF1 by Anchored Multiplex PCR in Malignant Peripheral Nerve Sheath Tumor,” Pathology, Research and Practice 216, no. 1 (2020): 152760, 10.1016/j.prp.2019.152760. 31812440

[13] Y. Tsuda , L. Zhang , P. Meyers , W. D. Tap , J. H. Healey , and C. R. Antonescu , “The Clinical Heterogeneity of Round Cell Sarcomas With EWSR1/FUS Gene Fusions: Impact of Gene Fusion Type on Clinical Features and Outcome,” Genes, Chromosomes & Cancer 59, no. 9 (2020): 525–534, 10.1002/gcc.22857. 32362012 PMC7372700

[14] R. Perret , V. Velasco , S. Le Guellec , J. M. Coindre , and F. Le Loarer , “The SS18‐SSX Antibody Has Perfect Specificity for the SS18‐SSX Fusion Protein: A Validation Study of 609 Neoplasms Including 2 Unclassified Tumors With SS18‐Non‐SSX Fusions,” American Journal of Surgical Pathology 45, no. 4 (2021): 582–584, 10.1097/PAS.0000000000001628. 33234877

[15] A. Hodgson , R. Benayed , M. Ladanyi , et al., “Uterine Sarcomas With a Novel SS18‐VEZF1 Fusion‐Another Neoplasm in the Uterine Myxoid Neoplasm Differential Diagnosis,” abstract presented at the 110th Annual Meeting USCAP, March 13–18, 2021.

[16] P. Dundr , J. Hojný , J. Dvořák , et al., “The Rare Gynecologic Sarcoma Study: Molecular and Clinicopathologic Results of A Project on 379 Uterine Sarcomas,” Laboratory Investigation 105, no. 5 (2025): 104092, 10.1016/j.labinv.2025.104092. 39921027

[17] P. S. Shukla and K. Mittal , “Inflammatory Myofibroblastic Tumor in Female Genital Tract,” Archives of Pathology & Laboratory Medicine 143, no. 1 (2019): 122–129, 10.5858/arpa.2017-0575-RA. 29965784

[18] F. Micci , S. Heim , and I. Panagopoulos , “Molecular Pathogenesis and Prognostication of “Low‐Grade” and “High‐Grade” Endometrial Stromal Sarcoma,” Genes, Chromosomes & Cancer 60, no. 3 (2021): 160–167, 10.1002/gcc.22907. 33099834 PMC7894482

[19] K. M. Devins , R. P. Mendoza , M. Shahi , et al., “Low‐Grade Endometrial Stromal Sarcoma: Clinicopathologic and Prognostic Features in a Cohort of 102 Tumors,” American Journal of Surgical Pathology (2025), 10.1097/PAS.0000000000002428. 40434060

[20] M. S. Moura , J. Costa , V. Velasco , et al., “Pan‐TRK Immunohistochemistry in Gynaecological Mesenchymal Tumours: Diagnostic Implications and Pitfalls,” Histopathology 84, no. 3 (2024): 451–462, 10.1111/his.15082. 37988282

[21] S. Chiang , P. Cotzia , D. M. Hyman , et al., “NTRK Fusions Define a Novel Uterine Sarcoma Subtype With Features of Fibrosarcoma,” American Journal of Surgical Pathology 42, no. 6 (2018): 791–798, 10.1097/PAS.0000000000001055. 29553955 PMC6764747

[22] M. Michal , A. Agaimy , S. Croce , et al., “PLAG1‐Rearranged Uterine Sarcomas: A Study of 11 Cases Showing a Wide Phenotypical Spectrum Not Limited to Myxoid Leiomyosarcoma‐Like Morphology,” Modern Pathology 27, no. 9 (2024): 100552, 10.1016/j.modpat.2024.100552. 38942115

[23] A. Agaimy , B. A. Clarke , D. L. Kolin , et al., “Recurrent KAT6B/A::KANSL1 Fusions Characterize a Potentially Aggressive Uterine Sarcoma Morphologically Overlapping With Low‐Grade Endometrial Stromal Sarcoma,” American Journal of Surgical Pathology 46, no. 9 (2022): 1298–1308, 10.1097/PAS.0000000000001915. 35575789 PMC9388494

[24] A. Momeni‐Boroujeni , K. Mullaney , S. E. DiNapoli , et al., “Expanding the Spectrum of NR4A3 Fusion‐Positive Gynecologic Leiomyosarcomas,” Modern Pathology 37, no. 5 (2024): 100474, 10.1016/j.modpat.2024.100474. 38508521

[25] P. Argani , B. Boyraz , E. Oliva , et al., “GLI1 Gene Alterations in Neoplasms of the Genitourinary and Gynecologic Tract,” American Journal of Surgical Pathology 45, no. 5 (2022): 677–687, 10.1097/PAS.0000000000001844. PMC9018467 34907995

