# Supplementary material for: Uterine Myxoid Mesenchymal Tumor With a Novel SS18::VEZF1 Gene Fusion, Lacking Worrisome Histological Features
Source: Genes Chromosomes Cancer. 2025 Aug 28;64(8):e70079. doi: 10.1002/gcc.70079 (PMC12394789; doi:10.1002/gcc.70079)
Supplement: Supplementary file 1 — Table S1: Supporting Information. [file GCC-64-e70079-s001.pptx]

## Slide 1
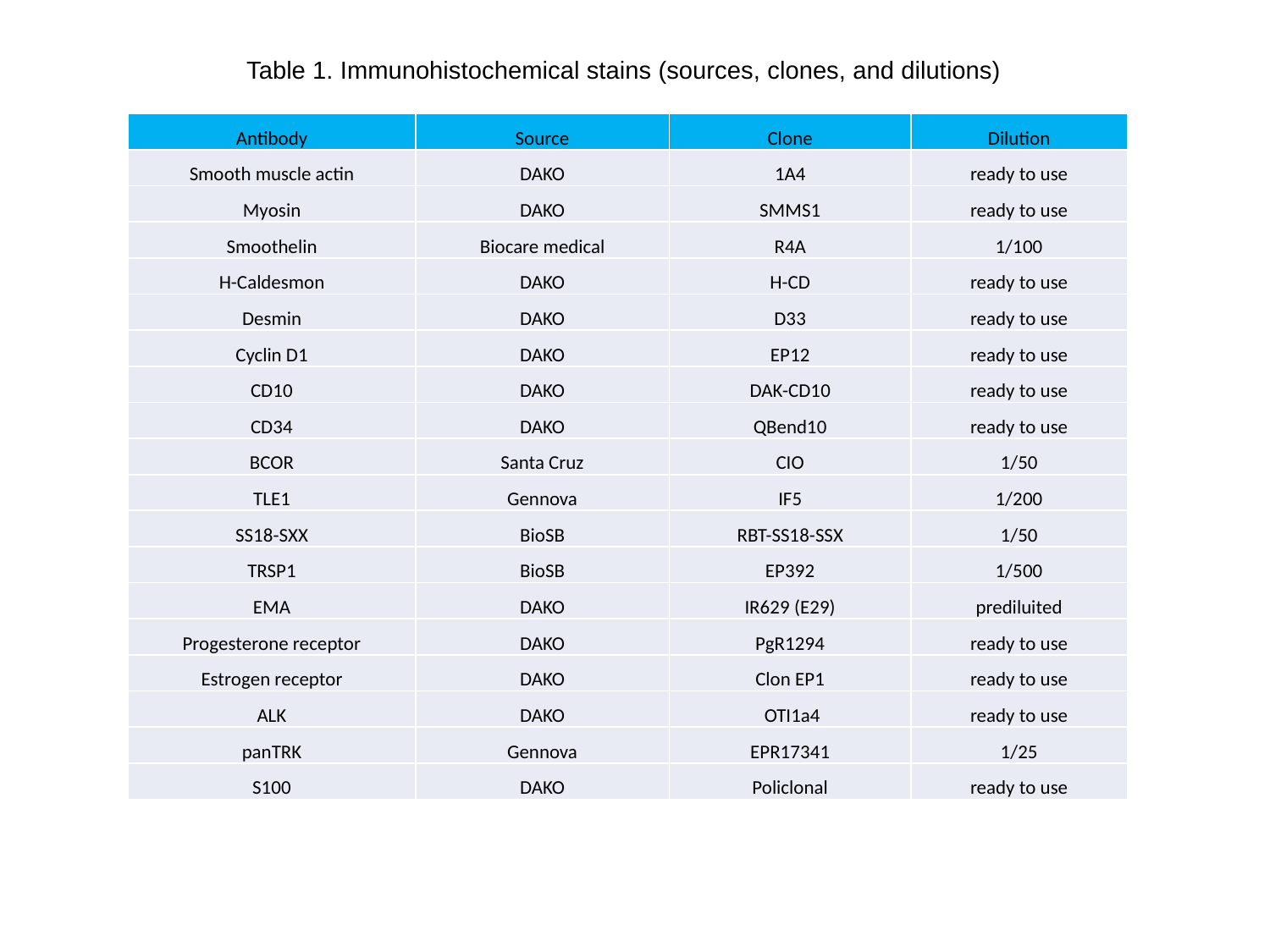

Table 1. Immunohistochemical stains (sources, clones, and dilutions)
| Antibody | Source | Clone | Dilution |
| --- | --- | --- | --- |
| Smooth muscle actin | DAKO | 1A4 | ready to use |
| Myosin | DAKO | SMMS1 | ready to use |
| Smoothelin | Biocare medical | R4A | 1/100 |
| H-Caldesmon | DAKO | H-CD | ready to use |
| Desmin | DAKO | D33 | ready to use |
| Cyclin D1 | DAKO | EP12 | ready to use |
| CD10 | DAKO | DAK-CD10 | ready to use |
| CD34 | DAKO | QBend10 | ready to use |
| BCOR | Santa Cruz | CIO | 1/50 |
| TLE1 | Gennova | IF5 | 1/200 |
| SS18-SXX | BioSB | RBT-SS18-SSX | 1/50 |
| TRSP1 | BioSB | EP392 | 1/500 |
| EMA | DAKO | IR629 (E29) | prediluited |
| Progesterone receptor | DAKO | PgR1294 | ready to use |
| Estrogen receptor | DAKO | Clon EP1 | ready to use |
| ALK | DAKO | OTI1a4 | ready to use |
| panTRK | Gennova | EPR17341 | 1/25 |
| S100 | DAKO | Policlonal | ready to use |
